# Femoral Vein Occlusion or Stenosis Using a Suture-Mediated Vascular Closure Device After Catheter Ablation

**DOI:** 10.1016/j.jaccas.2022.03.029

**Published:** 2022-05-18

**Authors:** Hirofumi Arai, Takatoshi Shigeta, Yuichiro Sagawa, Atsuhito Oda, Koji Sudo, Karina Hara, Mitutoshi Asano, Tsukasa Shimura, Hidetoshi Suzuki, Manabu Kurabayashi, Hideki Arima, Satoshi Itoh, Masahiko Goya, Tetsuo Sasano, Yasuteru Yamauchi

**Affiliations:** a Department of Cardiology, Yokohama City Minato Red Cross Hospital, Yokohama, Kanagawa, Japan; b Department of Cardiovascular Surgery, Yokohama City Minato Red Cross Hospital, Yokohama, Kanagawa, Japan; c Department of Cardiovascular Medicine, Tokyo Medical and Dental University, Tokyo, Japan

**Keywords:** atrial fibrillation, catheter ablation, femoral vein occlusion, femoral vein stenosis, suture-mediated vascular closure device

## Abstract

A suture-mediated vascular closure device is useful for hemostasis of the femoral vein after catheter ablation; however, venous complications remain unclear. We encountered 2 cases of femoral vein occlusion and stenosis using a suture-mediated vascular closure device. Both patients underwent surgical repair and recovered venous flow. (**Level of Difficulty: Intermediate.**)

## Central Illustration

**Figure:**
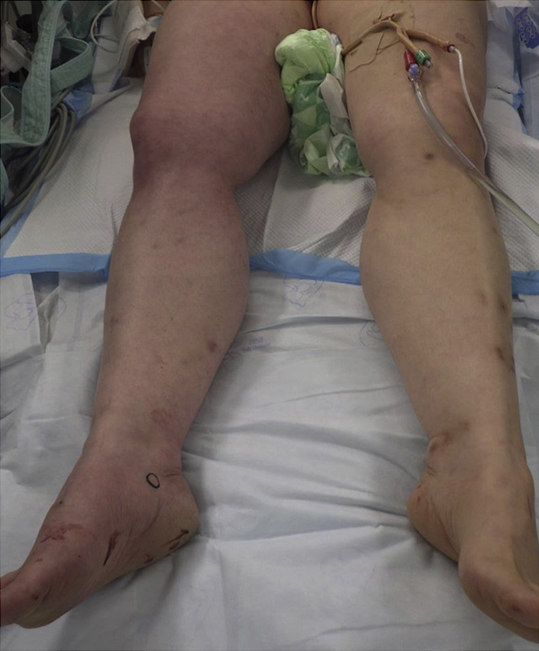

Catheter ablation of atrial fibrillation is a common ablation procedure. However, because of body movement restrictions due to the large sheaths used, overnight observation is needed for hemostasis of the femoral vein following this procedure. Suture-mediated vascular closure devices have been reported to achieve more hemostasis and to reduce hemostasis time.^1^ Arterial complications have previously been reported; however, venous complications due to a suture-mediated vascular closure device have not been well elucidated.

**Learning Objectives**

• To identify femoral vein occlusion or stenosis due to a suture-mediated vascular closure device.
• To understand the mechanism of femoral vein complications induced due to a suture-mediated vascular closure device.
• To treat femoral vein complications considering the underlying mechanism.

## Case 1

**Figure 1:**
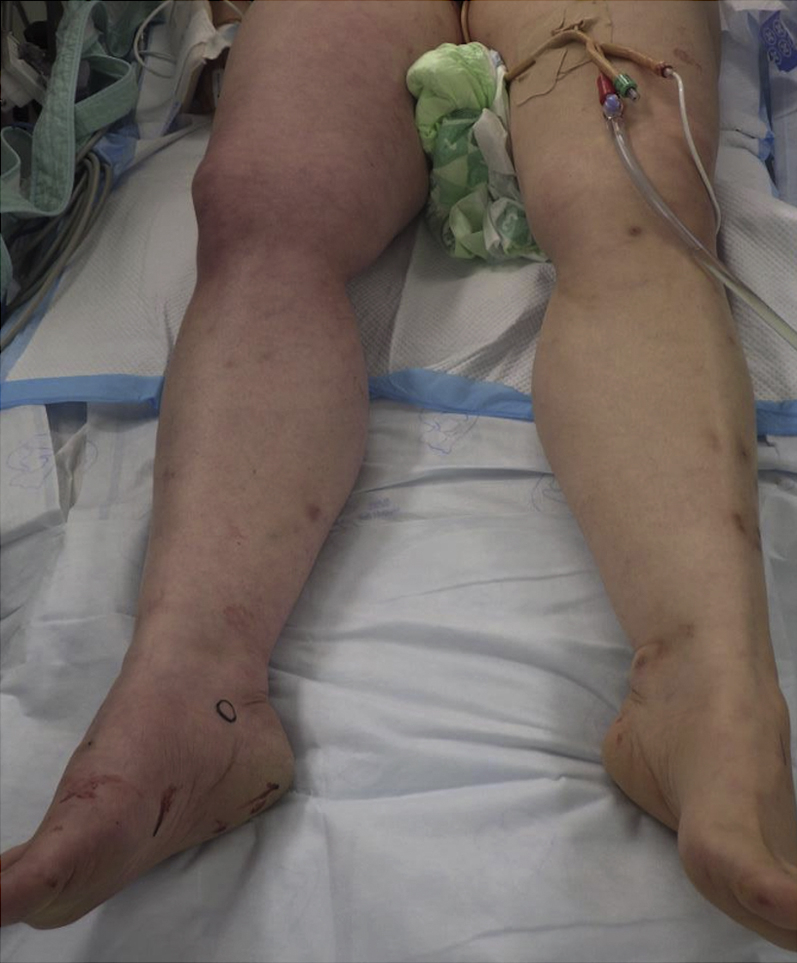
Gross Appearance of the Right Lower Extremity Preoperatively A purplish-red change in color to the right lower extremity is observed due to right femoral vein occlusion.

**Figure 2:**
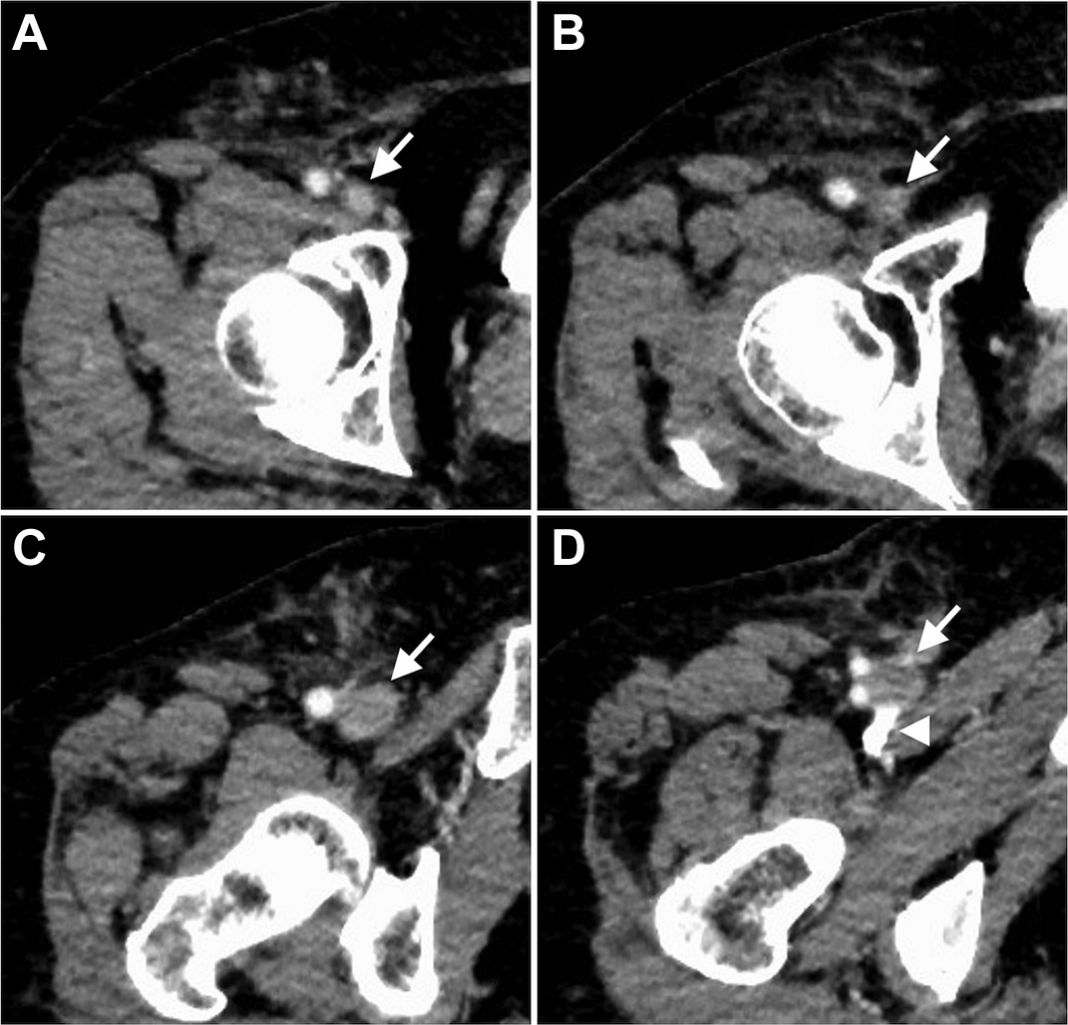
Contrast-Enhanced Computed Tomography of the Right Femoral Vein Occlusion The right femoral vein **(arrow)** is shown in a proximal **(A)** to distal direction **(D)**. Contrast enhancement is observed in the proximal side **(A)**, but it is unclear close to the puncture site **(B)**. The distal side is dilated **(C)** and contrast retention is observed **(D, arrowhead)**.

**Figure 3:**
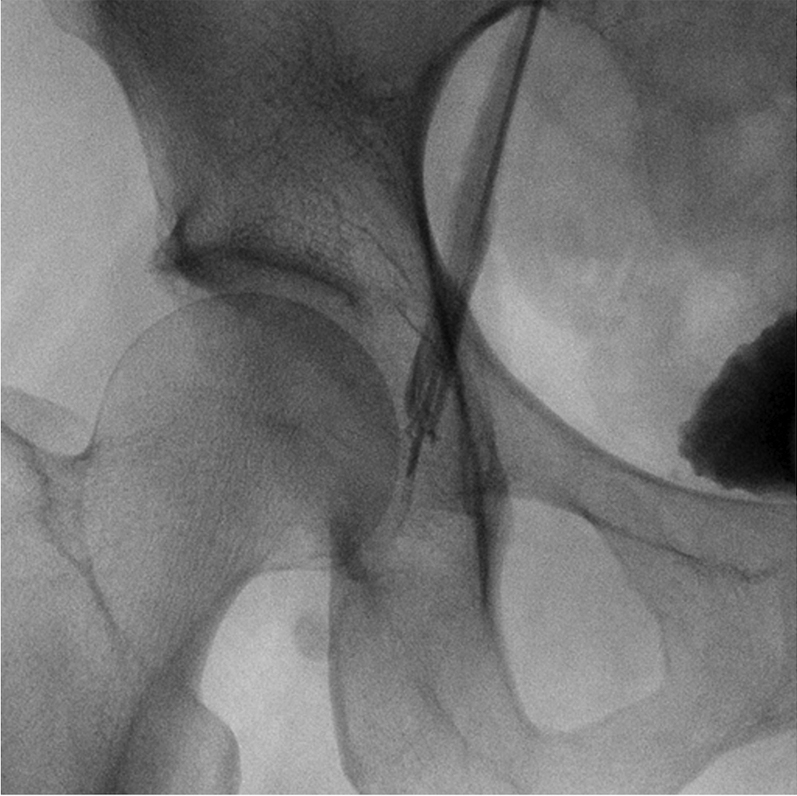
Venogram of the Right Femoral Vein Occlusion A venogram clarified the right femoral vein occlusion.

**Figure 4:**
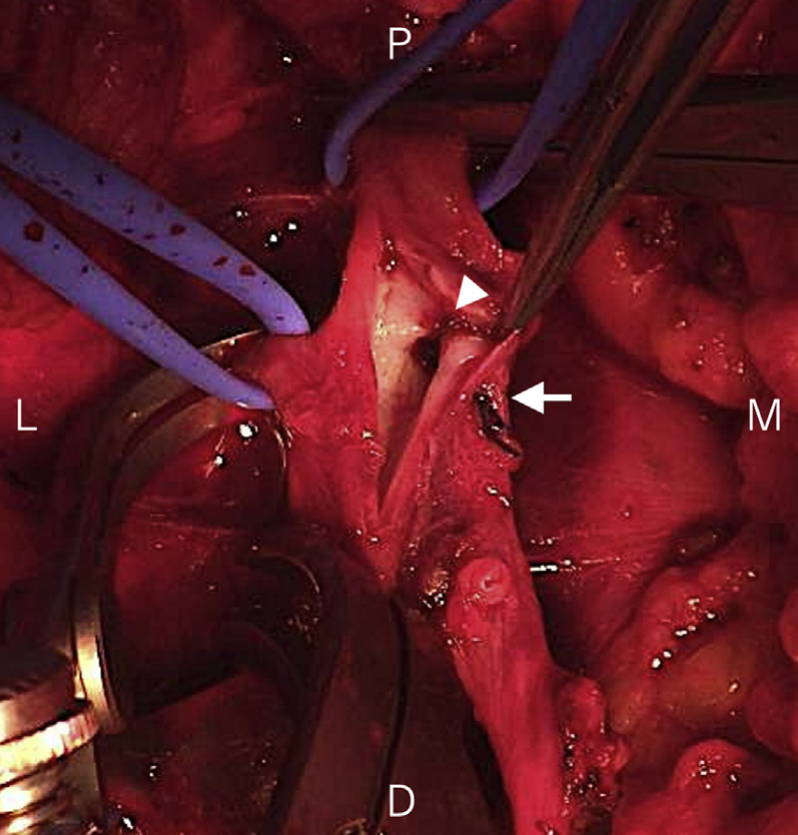
Posterior Wall Ligation With the Suture We incised the lateral side of the suture **(arrow)** and observed the inner lumen of the right femoral vein. The posterior wall was ligated using a suture **(arrowhead)**. D = distal; L = lateral; M = medial; P = proximal.

**Figure 5:**
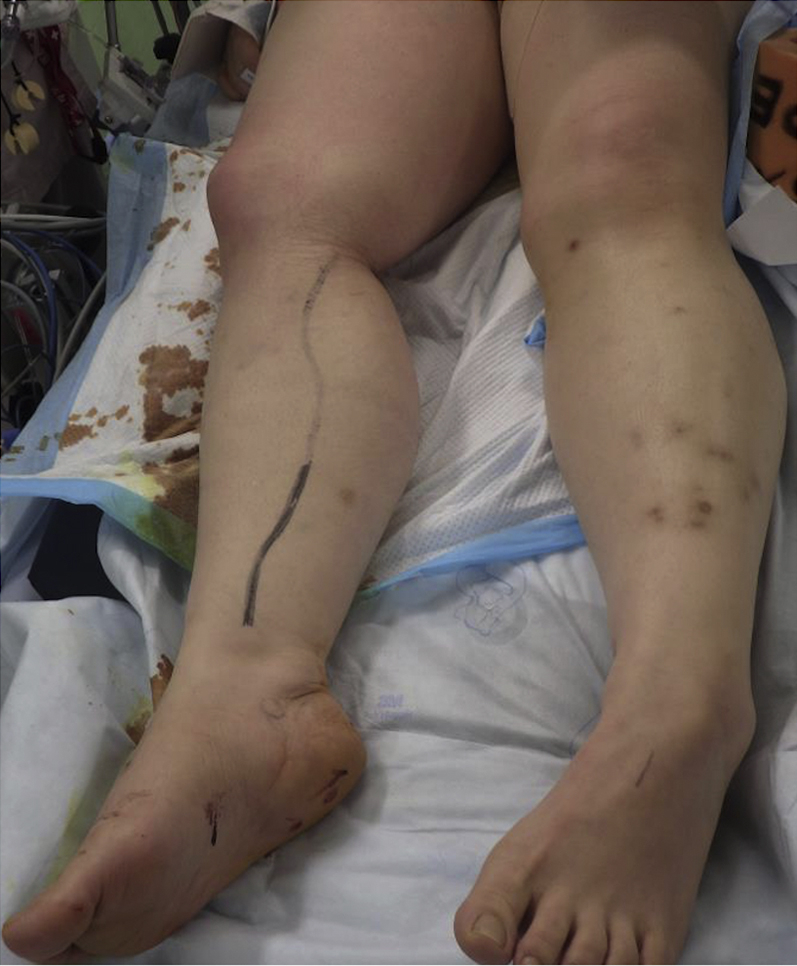
Gross Appearance of the Right Lower Extremity Postoperatively Color change to the right lower extremity improved postoperatively.

A 54-year-old woman with long-standing persistent atrial fibrillation and a decreased ejection fraction underwent catheter ablation. We punctured the right femoral vein in 2 places without ultrasonography guidance and inserted a Perclose ProGlide (Abbott) to apply the pre-close technique. We deployed needles at a 30° clockwise rotation for each puncture site before catheter insertion. A 15-F and an 8.5-F sheath were then inserted, and catheter ablation was performed. Postablation, the 15-F sheath was removed and hemostasis was achieved with a pre-closed suture but a similar result was not achieved with the 8.5-F sheath, and 2 additional sutures were required before hemostasis was achieved. We subsequently observed a purplish-red color change to the right lower extremity (Figure 1). Contrast-enhanced computed tomography showed retention of the right femoral vein blood flow and venous dilatation of the distal side of the puncture site (Figures 2A to 2D). A venogram showed right femoral vein occlusion (Figure 3). Three hours after hemostasis, we operated and found 2 of the 3 8.5-F sheath sutures had ligated the posterior wall of the femoral vein (Figure 4). After suture release, venous blood flow and right lower extremity color improved (Figure 5). The patient was discharged 6 days postoperatively.

## Case 2

**Figure 6:**
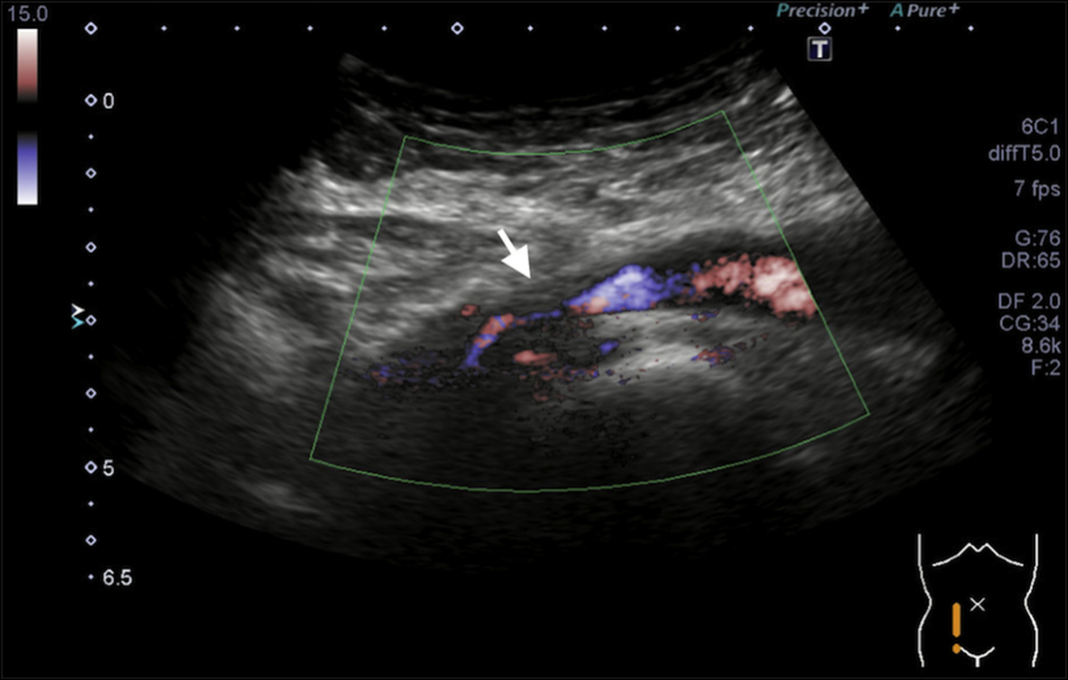
Ultrasonography of the Right Femoral Vein Stenosis The ultrasound scan indicated suspected right femoral vein stenosis **(arrow)**.

**Figure 7:**
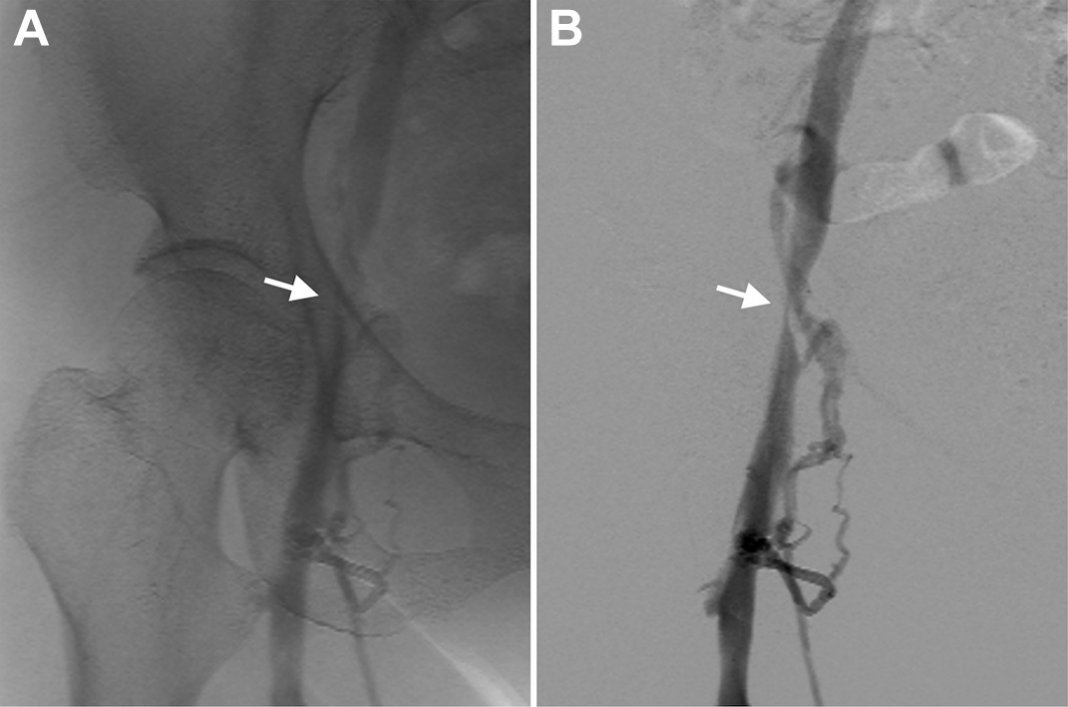
Venogram of the Right Femoral Vein Stenosis Venogram of digital angiography **(A)** and digital subtraction angiography **(B)** showing right femoral vein stenosis **(arrows)**.

**Figure 8:**
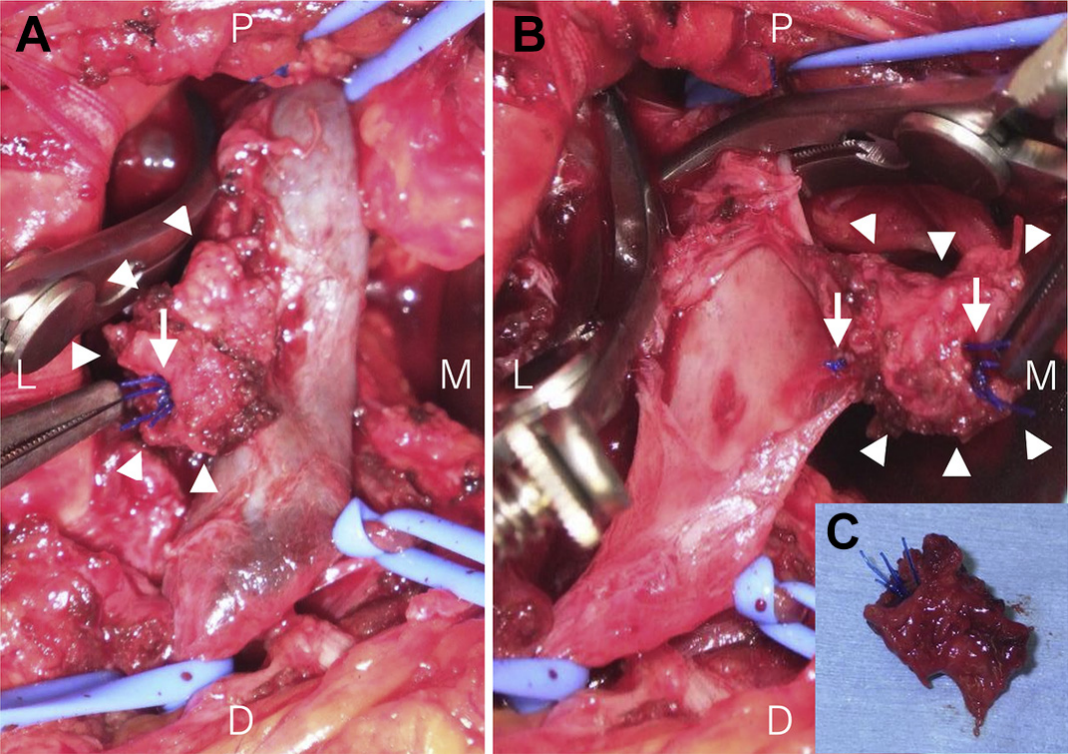
Abnormal Tissue Proliferation and Adhesion Tissue proliferation and adhesion **(arrowheads)** are observed around the suture **(arrows) (A)** An incision made to the lateral side of the suture showed the venous lumen, with no evidence of posterior wall ligation **(B)** Resected adhesive tissue is shown **(C)**. D = distal; L = lateral; M = medial; P = proximal.

A 77-year-old woman with symptomatic paroxysmal atrial fibrillation underwent catheter ablation. We punctured the right femoral vein in 3 places. Despite ultrasonography guidance, puncturing the vein was challenging, with multiple punctures applied. We performed a pre-close technique for each puncture site. One 11.5-F sheath and 2 8.5-F sheaths were inserted, and catheter ablation was performed. Post-procedure, we removed the 11.5-F sheath and attempted to close the puncture site with a pre-closed suture, which broke while being tightened. Another suture was added to achieve hemostasis. The remaining 2 sheaths were removed uneventfully. The patient was discharged 2 days after the ablation but presented to the emergency department with right lower extremity edema on postoperative day 13. No deep vein thrombosis findings were observed using contrast-enhanced computed tomography, and diuretic administration did not improve the edema. Right femoral vein stenosis was suspected on ultrasonography (Figure 6), and confirmed on venography (Figures 7A and 7B) on postoperative day 30. Surgery was performed on postoperative day 45. We found tissue proliferation and adhesion around the sutures, and the sutures were removed. There was no posterior wall ligation (Figures 8A to 8C) and the patient was discharged 6 days later.

## Discussion

We encountered femoral vein occlusion and stenosis using a suture-mediated vascular closure device. Femoral artery occlusion^2^ or stenosis^3^^,^^4^ have previously been reported, but no studies have reported femoral vein occlusion or stenosis.

In case 1, femoral vein occlusion was induced due to posterior wall ligation. Two possible mechanisms of posterior wall ligation for patients with arterial complications have been reported involving using the device to trap the arterial branch and dissecting the posterior wall intima through pulling the device at a lower angle, especially in a small vessel.^3^ Branch trapping also may occur in patients with venous complications, but this was not observed concerning case 1. We found 2 holes in the posterior wall of the femoral vein that may have been due to venous puncture. We hypothesized that posterior wall ligation might occur if the posterior foot trapped these holes. Up to 2 valves have been observed within 10 cm proximal to the saphenofemoral junction,^5^ with the mean distance from the saphenofemoral junction to the first valve being 4 cm.^6^ Therefore, the valve could be a risk factor for posterior wall ligation if the device foot is caught in the valve. A lower device angle, a small vessel, and deep insertion of the device also could be risk factors because they may facilitate trapping of the branches, puncture holes, and valves. Puncture site ultrasound evaluation is important before tightening a suture to facilitate identifying trapping of these structures.

In case 2, no posterior wall ligation was found, but tissue proliferation and adhesion around the suture were present. Femoral vein compression due to proliferative adhesive tissue may induce femoral vein stenosis. In femoral artery stenosis, an inflammatory reaction and thrombus due to endothelial damage or foreign body reaction to the suture have been reported as possible causes of stenosis.^4^ In our patient with femoral vein stenosis, multiple punctures because of the difficulty of puncture may have promoted inflammation and resulted in tissue proliferation and adhesion.

The VASCADE device is another useful venous vascular closure device for femoral vein hemostasis postablation, with no venous occlusion or stenosis-related complications reported.^7^ A lower risk of venous occlusion or stenosis with the venous vascular closure device is possible, because of its simple structure and no requirement for sutures.

Surgery and angioplasty are treatment options for femoral artery occlusion or stenosis^2^, ^3^, ^4^; however, there is no established treatment for femoral vein complications. We undertook a surgical approach, as angioplasty may have posed challenges if posterior wall ligations had been presented. Femoral vein stenosis due to adhesive tissue with no posterior wall ligation can be treated using angioplasty; however, its efficacy remains unclear, as proliferative adhesive tissue surrounding the vein may complicate its use.

## Conclusions

Femoral vein occlusion or stenosis is a possible complication of suture-mediated vascular closure devices. Posterior wall ligation or compression due to proliferative adhesive tissue may induce complications. There are no established treatments; however, surgical repair may be a suitable method.

## Funding Support and Author Disclosures

The authors have reported that they have no relationships relevant to the contents of this paper to disclose.
